# An Experimental and Empirical Study on the Use of Waste Marble Powder in Construction Material

**DOI:** 10.3390/ma14143829

**Published:** 2021-07-08

**Authors:** Muhammad Sufian, Safi Ullah, Krzysztof Adam Ostrowski, Ayaz Ahmad, Asad Zia, Klaudia Śliwa-Wieczorek, Muhammad Siddiq, Arsam Ahmad Awan

**Affiliations:** 1 School of Civil Engineering, Southeast University, Nanjing 210096, China; safi@seu.edu.cn (S.U.); siddiq@seu.edu.cn (M.S.); 2 Faculty of Civil Engineering, Cracow University of Technology, 24 Warszawska Str., 31-155 Cracow, Poland; klaudia.sliwa-wieczorek@pk.edu.pl; 3 Department of Civil Engineering, Abbottabad Campus, COMSATS University Islamabad, Islamabad 22060, Pakistan; 4 School of Civil Engineering, Zhengzhou University, Zhengzhou 450001, China; asadzia005@gs.zzu.edu.cn; 5 MM Pakistan (Pvt) Limited, Lahore 54000, Pakistan; arsam.ahmad@mmpakistan.com

**Keywords:** marble waste, bricks, clay, compressive strength, marble powder, eco-friendly materials

## Abstract

Marble is currently a commonly used material in the building industry, and environmental degradation is an inevitable consequence of its use. Marble waste occurs during the exploitation of deposits using shooting technologies. The obtained elements most mainly often have an irregular geometry and small dimensions, which excludes their use in the stone industry. There is no systematic way of disposing of these massive mounds of waste, which results in the occurrence of landfills and environmental pollution. To mitigate this problem, an effort was made to incorporate waste marble powder into clay bricks. Different percentage proportions of marble powder were considered as a partial substitute for clay, i.e., 5–30%. A total of 105 samples were prepared in order to assess the performance of the prepared marble clay bricks, i.e., their water absorption, bulk density, apparent porosity, salt resistance, and compressive strength. The obtained bricks were 1.3–19.9% lighter than conventional bricks. The bricks with the addition of 5–20% of marble powder had an adequate compressive strength with regards to the values required by international standards. Their compressive strength and bulk density decreased, while their water absorption capacity and porosity improved with an increased content of marble powder. The obtained empirical equations showed good agreement with the experimental results. The use of waste marble powder in the construction industry not only lowers project costs, but also reduces the likelihood of soil erosion and water contamination. This can be seen to be a crucial factor for economic growth in agricultural production.

## 1. Introduction

Marble is a crystalline metamorphic rock that can be formed into different shapes and sizes for flooring, monumental and decorative purposes. According to a survey, the demand for marble stone worldwide hit 816 million m^3^ in 2016. In 2019, the global demand for marble was estimated at USD 55,420 million, and it is projected to hit USD 68,790 million by the end of 2026. This is an annual increase of 3.1% [1]. Pakistan’s marble and granite deposits are projected to be equal to 300 billion tons. In addition, the country’s gross monthly marble output is approximately 1 million tons, with 2000 processing units and 1225 quarries in service. As a consequence, in a country like Pakistan, where the marble industry is huge, pollution is a major concern. Pakistan has a range of marble processing plants, where marble waste is generated on a regular basis. This marble waste, in the form of slurry and mud, is discarded in open spaces (without adequate disposal) and has harmful environmental consequences [2,3]. The powdered form of marble stone decreases soil fertility by increasing alkalinity, endangering plants, and wreaking havoc on the environment. There is also a major loss in fauna and flora, as the slurry of marble accumulation impacts foliage and plant leaves, possibly drying out already grown trees and bushes [4,5,6]. The valuable powdered form is discarded by the marble industry, which has an impact on financial development and even results in environmental pollution [7]. Similarly, the high production of clay bricks in Pakistan is also causing soil degradation. As a consequence, the use of marble wastes as an alternate material in the manufacturing of bricks may be both inexpensive and beneficial for the atmosphere.

### 1.1. The Use of Various Types of Waste Materials in Clay Bricks

Many studies have used various wastes as additives in the manufacturing of clay bricks in order to test their various properties. The use of waste materials for construction purposes can be helpful in reducing the risk of shortages in natural resources and for improving the environment [8,9,10]. The addition of different solid wastes for the production of fired clay bricks were tested. As a result, bricks with improved properties, such as thermal conductivity, density, porosity, and water absorption, were obtained. According to Kadir A.A. and Mohajerani A., brick is a material with a relatively high mechanical strength, which can be recycled and reused in the production of other construction materials [11]. Muñoz Velasco P. et al. carried out a study regarding the use of various sorts of wastes in fired clay bricks and found the use of waste in clay bricks was eco-friendly and, in some cases, improved their properties [12]. Some studies related to the use of various types of wastes and low-cost materials are presented below.

### 1.2. The Use of Rice Husk Ash

Fernando P.R. compared the physical and chemical properties of rice husk ash bricks with traditional bricks and found that the addition of 5% of rice husk ash improves the compressive strength and water absorption properties of the bricks [13]. Rao B.J. used different wastes, such as fly ash, rice husk, sawdust, and bagasse in order to make clay bricks. Experimental tests were carried out to check the physical and mechanical properties of the resultant bricks. It was shown that the addition of waste materials to the brick mixture affects the consistency of freshly formed bricks [14].

### 1.3. The Use of Rice Fly Ash, Silica Fume, Wood Dust, Slags and Dry Grass

Kadir A.A. and Sarani N.A. assessed numerous waste materials, such as fly ash, rubber, limestone, wood dust, and sludge. They noticed that such waste had a beneficial effect on the manufactured lightweight bricks, and also enhanced the thermal conductivity of fired clay bricks [15]. Zhang L. presented a review of the research work concerning the use of waste materials for the production of bricks. Many waste materials, including fly ash and slag, as well as different methods, were studied with regards to the production of bricks [16]. Baspinar M.S. et al. found that silica fume in fired clay bricks improves the strength and efflorescence properties of the bricks. In addition, the potency of silica fume depends on the temperature of the fire [17]. Abbas S. et al. prepared bricks using clay and up to 25% of fly ash. It was found that the compressive strength of the bricks with the addition of fly ash was lower than the bricks without fly ash. In contrast to the clay bricks, the fly ash bricks were lighter and had less efflorescence [18]. Phonphuak N. prepared clay bricks with different percentages of dry grass and tested them in order to find out about the different properties of the resultant product. As a result, it was revealed that the bulk density and compressive strength of the bricks decreased with an increase in the content of dry grass [19].

### 1.4. The Use of Wood Ash, Marble Powder, and Other Types of Waste Powders

Oorkalan A. et al. used different proportions of waste products of wood ash, ceramic powder, and marble powder in clay bricks. Compressive strength and water absorption tests were conducted. It was concluded that the raw materials were useful and increased the brick’s compressive strength and durability [20]. Ngayakamo B.H. et al. prepared fired clay bricks by using granite and eggshell powder in order to reduce environmental pollution. The obtained findings showed that the fired clay bricks made of 20% granite and 10% eggshell powder had the best performance [21].

### 1.5. The Use of Glass Wastes

Incorporating glass waste into fired clay bricks is an excellent way to develop eco-friendly bricks and other enhanced materials for the construction industry. Xin Y. et al. manufactured fired clay bricks with a partial addition of glass waste. They discovered that as the glass content rose and the particle size decreased, the compressive strength of the fired clay bricks improved significantly [22]. Mobili A. et al. used glass-reinforced plastic dust as a partial replacement for clay to produce fired clay bricks. The addition of GRP dust improved the water absorption capacity and decreased the effect of the firing of clay bricks [23]. Kazmi S. M. S. et al. used waste glass sludge to manufacture clay bricks and concluded that the bricks made of waste glass sludge achieved a higher compressive strength and flexural strength than the reference clay bricks. Moreover, adding waste glass sludge made the bricks lighter and increased their tolerance to efflorescence, sulphate attack, and freeze-thaw cycles [24].

### 1.6. The Use of Natural Fibers

It has been proven that some natural fibers can improve the strength properties of composite materials and also be used to produce cheap bricks from fired clay [25]. Kadir A.A. et al. made low-cost clay bricks with different amounts of coconut fiber in order to assess their physical and chemical properties. It was shown that the use of coconut fibers as an additive to the brick mixture allows this waste to be effectively used, and at the same time, bricks with good quality to be obtained [26]. Kadir A. A. et al. investigated the physical and mechanical properties of palm kernel shells to be used as a substitute for clay in fired clay bricks. Palm kernel husk was considered to be a waste that could be used in the production of bricks. This is because, with its addition, a product of acceptable quality was obtained [27].

### 1.7. The Use of Marble Dust in Clay Bricks

An experimental study was conducted on marble waste (in different proportions) as an additive to bricks. It was proven that the marble waste had a good impact on the chemical, physical, and mechanical strength of the resultant bricks [4,28,29]. Considering the mechanical properties of bricks containing wastes, it has been shown that the addition of marble powder to a mixture has no prominent impact on their load-bearing capacity [30]. Kathiresan M. et al. used marble sludge powder as a partial replacement for clay in order to prepare modified clay bricks with enhanced durability and strength properties. It was discovered that the use of marble sludge powder in brick manufacturing results in safe and environmentally sustainable recycled materials [31]. It is worth mentioning that Seghir N.T. et al. used waste marble powder as a partial replacement for cement in mortar in order to assess the properties of the resultant product, i.e., compressive strength, density, and apparent porosity. The obtained results showed that the addition of waste marble powder reduced the density and compressive strength of the elements, while at the same time improving their porosity [32]. Ramachandran G. et al. used granite and marble wastes in fly ash bricks to determine their strength properties and stated that the use of such wastes as binding materials is helpful in minimizing the risk of pollution [33]. Rehman W. et al. stated that marble waste could be used to prepare concrete bricks. Furthermore, it was shown that the compressive strength of bricks depends on the mutual mass proportions of marble dust, sand, and cement [34].

### 1.8. The Use of Marble Powder in Cement-Based Materials

Many researchers have used marble waste and brick waste in different types of concrete and cement mortar. It has been determined that concrete made with the addition of waste marble powder can be ecological. This is consistent with sustainable development strategies, which are now a global trend [35,36]. According to Shah M.U. et al., ultrafine brick waste has a high degree of pozzolanic ability and can be used as an addition to cement. In paper [37], it was shown that the mechanical properties of cement paste containing 5% and 10% of waste burnt brick powder were higher than for the reference mixture containing no waste. According to [38], the use of marble powder improved carbonation and water resistance, decreased shrinkage strain, and reduced the content of cement. Sadek M. D. et al. used waste marble and granite powders as mineral additives in self-compacting concrete. According to the researchers, waste powders should be used as mineral additives in self-compacting concrete to improve its performance [39]. Rodrigues R. et al. investigated the mechanical properties of concrete with different marble sludge-to-cement substitution ratios. Concrete containing marble sludge had lower mechanical properties than the reference samples [40]. The effect of marble powder as a partial replacement for cement on the mechanical properties and toughness of high-performance concrete was examined by Talah A. et al. It was found, when compared to the reference concrete, that marble powder is a good additive for the manufacturing of concrete with improved mechanical properties [41,42]. Elmaghraby M. S. and Ismail A. I. M. explored the chemical, mechanical, and mineralogical properties of the waste kaolinitic sand used to prepare concrete. According to their test results, some mixtures can be used in industrial furnaces at 1500 °C in order to produce concrete [43]. In summary, it is worth emphasizing that the use of waste marble powder for the production of many types of concrete would be beneficial for the natural environment due to the positive aspect of waste disposal. Thus, it is possible to obtain good concrete properties in terms of workability, strength, permeability, and microstructural performance [44].

## 2. Research Significance

The Earth’s ecosystems are being destroyed, and there is a continuing increase in water pollution, bad air quality, and ground contamination. Pollution is not only a health issue, but also a major obstacle for sustainability. To minimize this challenge, researchers and engineers need to be more focused on the effective use of waste materials in the construction industry. The use of waste materials is one of the most critical steps of sustainability, because it helps to minimize the impact of environmental degradation, save renewable resources, lower the overall cost of building projects, and bring economic value to waste materials. It is more and more clear that advances in the production of waste derived materials benefit society. Marble and granite factories generate a large volume of wastes, including sludge and other residues, which pose a serious threat to the environment by polluting soil and water. In this paper, industrial waste in the form of marble powder was used as the base material for producing bricks. These bricks are inexpensive, offer good compressive strength, and are lightweight. The current research project aims to identify the waste-related commodity arrangements and treatment scenarios that are suitable for the production of marble powder-based slurry for sustainable bricks. This study assesses the properties of the final product after incorporating waste marble powder. As a result of this study, the use of waste marble powder in the construction industry might lead to a sustainable and environmentally friendly material with better properties. The key points of this study were to examine the effects of waste marble powder on the different properties of sustainable marble clay bricks, i.e., water absorption ability, bulk density, apparent porosity, efflorescence, and compressive strength. When optimizing the composition of waste marble bricks, it is possible to minimize the risk of environmental contamination by reducing the amount of marble waste. The use of waste marble powder would be advantageous due to the use of wastes, as well as for obtaining good strength and low-cost bricks beneficial for sustainable construction.

## 3. Materials and Experimental Methodology

**Figure 1:**
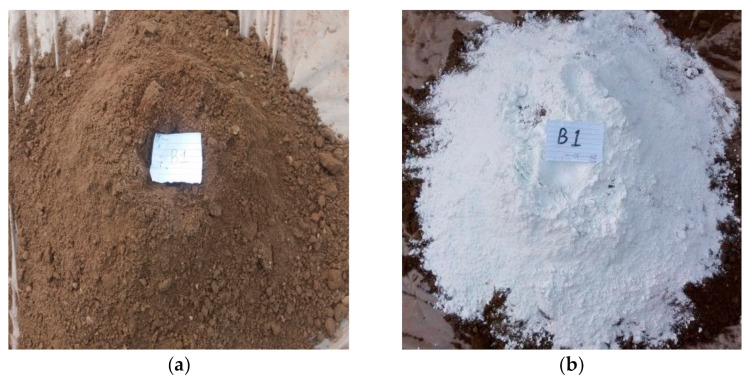
View of: (**a**) clay sample, and (**b**) mixing of clay and marble dust.

**Figure 2:**
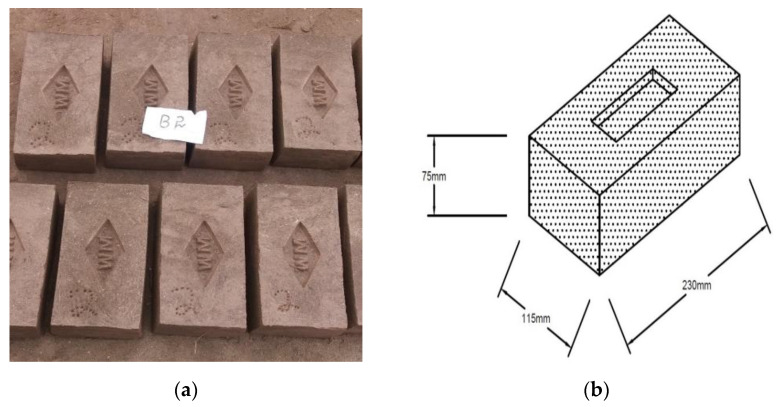
View of: (**a**) prepared marble clay bricks, and (**b**) standard brick size.

The materials used in this research were marble powder, which was collected from a local company in Peshawar, Pakistan, and clay from a kiln in the district of Peshawar, where the bricks were prepared. The type of soil in this region is clay loam containing 30.9% sand, 37.8% silt and 31.3% clay [45]. The research methodology of this project included the preparation of raw materials, the dosing of a mixture of marble powder and clay waste, the formation of bricks, the drying process, the firing and cooling process, and laboratory tests. To manufacture marble clay bricks, many sites were visited, with one of the finest kilns in the region being selected. The place where the soil was collected was first cleaned and excavated to a depth of five feet. The required quantity of earth was then taken, cleaned from stones, and converted into powder form using a small earth crushing roller available in the kiln. The pulverized clay was sprinkled with water and kept in the conditions of an open laboratory space for 24 h before it was used, as shown in Figure 1a. The clay was prepared at the site and then manually mixed with different amounts of marble powder, see Figure 1b. The tempering process was carried out by adding the appropriate amount of water to the clay, which was then mixed thoroughly to make a homogenous mixture. The pressing of the mixture was done with the help of peoples’ feet. The mixing time was about 15 min. A temperature of 25 ± 1 °C and humidity of 48 ± 2% was recorded during this work. The marble powder and clay proportions are given in Table 1. The steel molds were filled with tempered clay and pressed hard in order to appropriately fill the corners of the mold. An extra clay on the top surface of the molds was removed with the help of a wooden plank. The molds were then lifted up and properly shaped raw bricks were made on the ground, as shown in Figure 2a. Before the molding process, the ground was levelled, and sand was sprinkled over it. The molded bricks were dried using the hack drying method, i.e., the wet bricks were arranged in rows on their edges on slightly raised ground (hacks) in such a way that an appropriate space was kept between the rows for air and heat circulation. It was ensured that there was no sudden drying caused by direct exposure to the sun and wind, and a portable cover was provided to protect the bricks from rain. The naturally dried bricks were then taken for firing to a kiln called the ‘bull trench kiln’, the temperature of which was 1000–1100 °C. The burnt bricks were taken from the kiln and kept in an open place for cooling. A total of 105 brick samples were prepared; 15 samples for each marble proportion. All the samples were then safely deposited in a laboratory. The standard brick size available in Pakistan is shown in Figure 2b.

### 3.1. Energy Dispersive X-ray (EDX) Test of Marble Powder

**Figure 3:**
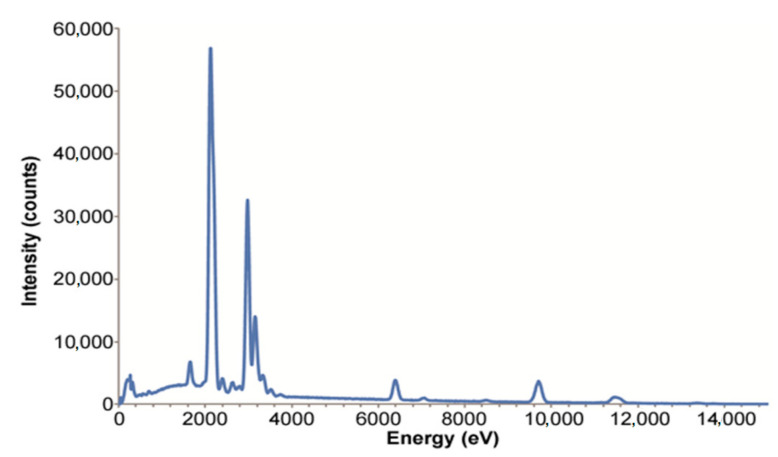
EDX test result.

To check the elements of marble powder, the energy dispersive X-ray test (EDX) was conducted. The contact area from which the X-rays were released was within the spectrum of 1 μm^3^ for SEM-EDX. X-ray photons were examined spectroscopically in order to obtain basic information about the sample. Various images of regions were taken throughout the course of the EDX test, and the associated peaks for each element are displayed in Figure 3. The greatest density at the extreme peak at 2000 (eV) represents the oxides with the atomic value of 64.76%, while the second highest peak at about 3000 (eV) indicates the carbon element content with a value of 18.33%. Thirdly, the minor peaks seen at various energy levels, i.e., in the initial area until 1800 (eV), 6000 (eV) and 10,000 (eV), suggest a calcium content of about 16.71%. The lowest peaks imply a magnesium content of about 0.20%. The peak intensity of oxides was greater due to their preferred crystal orientation, while the other components’ peak intensity was lower due to their randomized crystal orientations. The values of elements measured in weight % and atomic % are given in Table 2. The first letter—C, O, Ca and Mg—denotes elements identified during the EDX examination, while the second letter, K, denotes the shell of the element. 

### 3.2. Water Absorption Test

**Figure 4:**
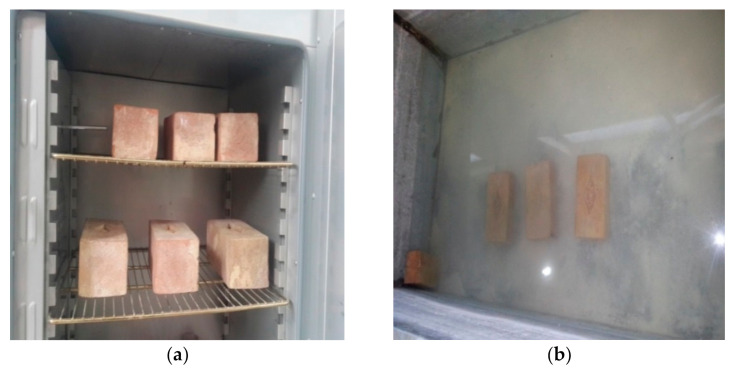
Water absorption test: (**a**) drying the samples in the oven, and (**b**) soaking the samples in water.

The water absorption tests were carried out on each of the groups of the analyzed brick samples. Three specimens of bricks from each mix group were tested. The ASTM C67-07 standard, 2007 [46] was followed. All the specimens of bricks were checked for loose debris and chips and were then wiped clean before the test. The specimens were dried in a ventilated oven at a temperature of 105 °C to 115 °C (Figure 4a). The specimens were cooled at room temperature and their weight was noted. The dried specimens were fully submerged in water from waterworks at a room temperature of 25 ± 1 °C for 24 h (Figure 4b). The specimens were then detached and wiped with a damp cloth and weighed again. 

### 3.3. Efflorescence Test

**Figure 5:**
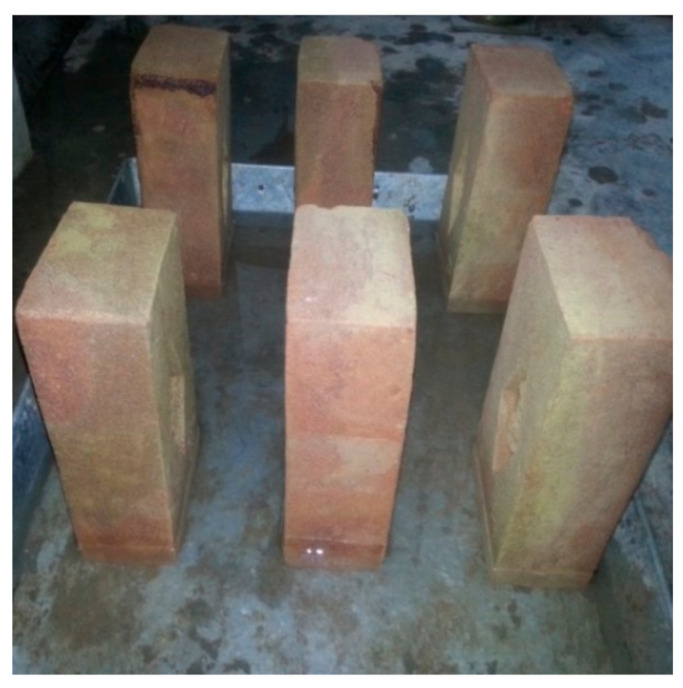
Efflorescence test.

The efflorescence test was performed on three brick samples taken from each mix proportion group, according to the specifications of the ASTM C67-07 standard, 2007 [46]. Thin flat-bottomed trays with appropriately distilled water were used to saturate the brick specimens. The brick specimens were vertically submerged in water up to 25 mm, as presented in Figure 5. The entire procedure took place in a well-ventilated room with a temperature of 25 ± 1 °C and a humidity of 48 ± 2%. This process continued until all the water in the trays had been absorbed by the brick specimens and the surplus water had evaporated. When the water had been absorbed and the brick appeared to be dry, a similar quantity of water was again placed in the trays and allowed to dry as before. The bricks were inspected for efflorescence after the second evaporation. The purpose of this test was to check the white crystalline salty deposits on the surface of the bricks. The tolerance of the bricks to the salt attack is determined based on the amount of salt efflorescence on their surfaces. The goal of this test was to see whether the surface of the marble powder bricks had a white crystalline salty coating. The creation of salt deposits on the surface of bricks is caused by the presence of water (moisture) in either the bricks or the surrounding environment, which dissolves the salts contained within the bricks and transports them to the bricks’ surface due to capillary action. When saltwater reaches the surfaces of the bricks, the water evaporates due to air, in turn leaving a fine white powdered salt layer. This indicates that the occurrence of efflorescence is dependent on the circumstances of exposure, the type of bricks (dense or porous), and the nature of the components employed in the production of the bricks.

### 3.4. Bulk Density Test

The bulk density test was conducted to find the tapped bulk density or bulk volume of the marble clay bricks according to the ASTM C134-95 standard [47]. The apparatus used in this test included a flat metal rule with a square at one end for measuring the brick samples, an oven for drying purposes, and a balance to weigh the brick samples. The three dimensions, i.e., length, width and thickness of each brick unit were measured carefully and noted. After the measurements, the brick samples were put inside the oven to dry at a controlled temperature of 110 °C for 2 h. The samples were then cooled inside the room and weighed. The air temperature was recorded to be 26 ± 1 °C. This procedure was repeated two times, and then the bulk density of the marble clay bricks was calculated by dividing the dry weight of the samples (in kg) by the samples’ volume (in m^3^).

### 3.5. Porosity Test

This test was performed according to the ASTM C67-07 [46] and ASTM C20-00 standards [48]. The brick specimens were dried at 110 °C in an oven and weighed after being cooled at room temperature. The specimens were then placed in distilled water and boiled for 2 h. Afterwards, they were cooled and weighed while still immersed in the water. After obtaining the suspended weight, the bricks were removed from the water immediately, blotted lightly with a moistened towel, and weighed. This procedure was done at an air temperature of 26 ± 1 °C.

### 3.6. Compressive Strength Test

**Figure 6:**
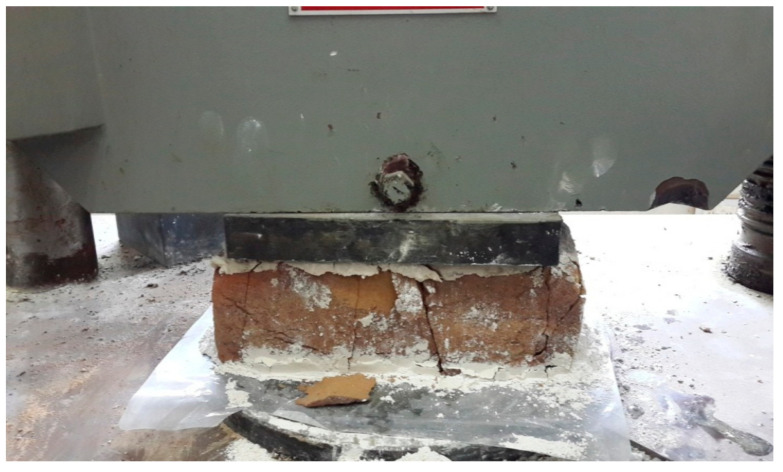
Compressive strength test.

To determine the compressive strength of the marble clay bricks, a compression test was performed according to the ASTM C67/C67M standard [48] by using a universal testing machine, as illustrated in Figure 6. Prior to the compression test, the unevenness of the bricks’ surfaces was removed in order to make the surfaces smooth and parallel. All the specimens were submerged in water at room temperature for 24 h. The specimens were then removed, and any surplus moisture was drained out. The frog and all the voids in the bed face were filled with plaster of Paris (1:2) and left to dry for the next 48 h. At the time of testing, the dimensions of each specimen were measured using a scale. The specimens were placed with their flat faces in a horizontal position, with the mortar filled face facing upwards, carefully positioned between the plates of the testing machine. The load was applied axially at a uniform rate of 14 N/mm^2^ per minute until failure occurred. A total of 3 specimens were tested for each proportion group, and the average of the obtained results was taken as the final compressive strength. The compression test was performed in a laboratory at a temperature of 26 ± 1 °C and a humidity of 48 ± 2%.

## 4. Test Results and Discussion

### 4.1. Water Absorption Test Results

**Figure 7:**
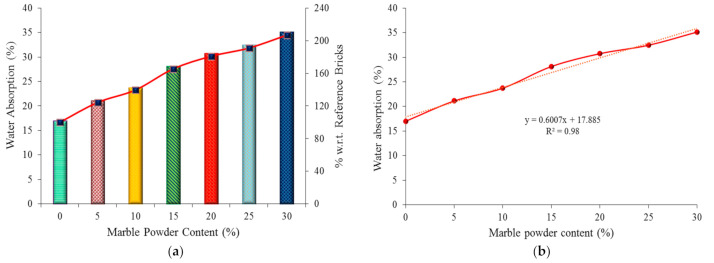
Graphical representation of the water absorption of the bricks: (**a**) experimental results, and (**b**) empirical results.

The results of the water absorption test are given in Figure 7a. Due to the fact that all the specimens were fully submerged in water from waterworks for the same amount of time (24 h), only water absorption percentages are shown on the graph’s Y-axis in the graphs. From the test results, it was noted that an increased content of marble powder raised the water absorption capacity of the bricks. The water absorption capacity of the marble powder bricks was respectively 4.13%, 6.73%, 11.13%, 13.75%, 15.46%, and 18.14% higher than the reference bricks (with a 0% content of the marble powder). The permissible maximum water absorption of a brick, according to ASTM specifications, is 20% of its total weight. However, it should be remembered that the maximum water absorption value depends on national requirements and may differ depending on the location. It should be noted that this value depends on the open porosity of bricks. Moreover, in recent years, the requirements for the maximum water absorption of bricks have become more stringent. For example, on the basis of PN-B 12011: 1997 [49] (recent requirements in Poland for the tenth class of bricks), PN-EN 771-1 [50] (actual requirements in Europe), and requirements for second-class bricks in Pakistan [51], the maximum water absorption is 24%, 22% and 25%, respectively. The waste marble clay bricks exceeded the value according to ASTM in all the tested group samples (i.e., with the addition of 5% to 30% of marble waste). An empirical equation was established between the water absorption capacity and the marble powder content, in order to compare the correlation coefficient value with the experimental results, as presented in Figure 7b. The experimentally calculated values in the current study were used to develop an empirical relation between the water absorption and marble waste percentage used in the bricks. Using the derived empirical equation, the expected water absorption for each percentage of marble waste was determined. Furthermore, the water absorption values for the samples with the addition of marble waste, which were obtained from the experimental and empirical equations, are compared in Figure 7b. The correlation coefficient (R^2^) value is equal to 98%, confirming a good compliance of the obtained empirical results with the experimental data.

### 4.2. Efflorescence Test Results

**Figure 8:**
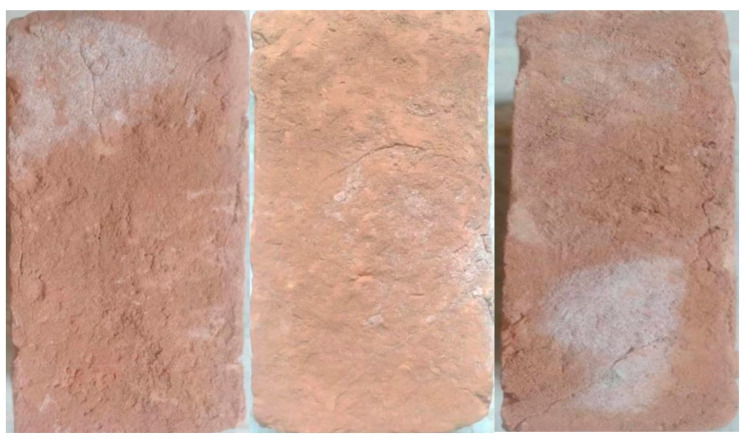
The effect of efflorescence.

From the conducted detailed observations and results, a slight efflorescence was seen, i.e., about 10–15% of the exposed areas of the B5 and B6 bricks were covered with a thin deposit of salt, as shown in Figure 8. The rest of the bricks had no perceptible deposits of efflorescence on their surfaces. It was shown that the addition of marble waste to bricks did not significantly affect the amount of salt efflorescence.

### 4.3. Bulk Density Test Results

**Figure 9:**
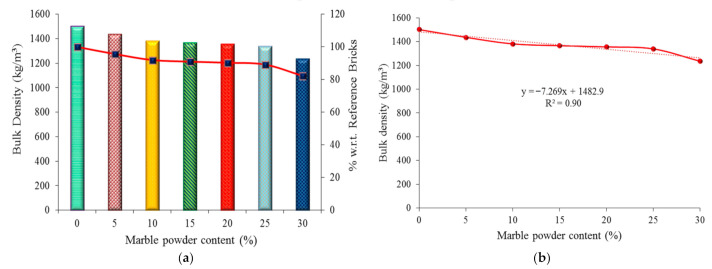
Graphical representation of bulk density: (**a**) experimental results, (**b**) empirical results.

The results of the bulk density of the clay bricks with the addition of waste marble powder are shown in Figure 9a. The bulk density of the waste marble clay bricks was lower by 4.5%, 8.7%, 9.9%, 10.74%, 12.1%, and 21.5%, respectively when compared with the reference bricks. The weight of waste marble powder bricks was measured, and it was found that the marble powder bricks are 1.3% to 19.9% lighter than the reference bricks that contain 0% of marble powder. The empirical equation is used for evaluating the bulk density with regards to the marble powder content used in the bricks (Figure 9b). The correlation coefficient (R^2^) value is equal to 90%, confirming a good compliance of the obtained empirical results with the experimental data.

### 4.4. Porosity Test Results

**Figure 10:**
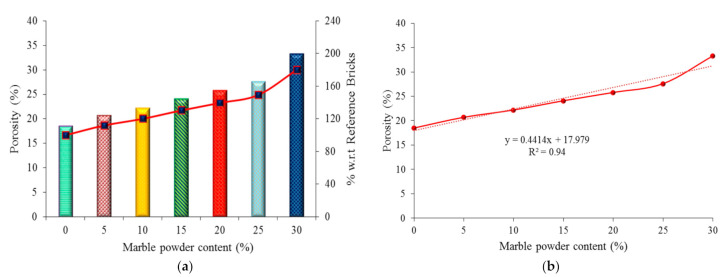
Graphical representation of porosity: (**a**) experimental results, (**b**) empirical results.

The obtained porosity results (in %) are presented in Figure 10a. The porosity ranged from 18.5% to 33.3%. The porosity of the marble powder bricks was higher when compared to the reference bricks. The porosity range increased with increasing marble powder content in the bricks. This is due to carbon dioxide (CO_2_) being released during the calcination process of the calcium carbonate (CaCO_3_). Figure 10b shows the empirical results of the porosity of the marble powder bricks. The correlation coefficient (R^2^) is equal to 94%, confirming a good compliance of the obtained empirical results with the experimental data.

### 4.5. Compressive Strength Test Results

**Figure 11:**
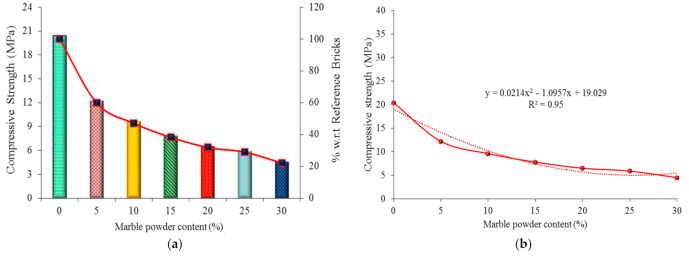
Graphical representation of compressive strength: (**a**) experimental results, and (**b**) empirical results.

The results of the compression test indicate that the compressive strength decreased with an increase in the marble powder content in the bricks. The highest compressive strength of 20.4 MPa was obtained by reference sample “A”, which contained a 0% marble powder content. The specimens of group “B1”, with a 5% marble powder content, achieved a compressive strength of 12.2 MPa. In turn, groups B2, B3, B4, B5, and B6, with a 10%, 15%, 20%, 25%, and 30% marble powder content, achieved a compressive strength of 9.6 MPa, 7.8 MPa, 6.5 MPa, 5.9 MPa, and 4.5 MPa, respectively. The compressive strength results are given in Figure 11a. The compressive strength of the waste marble clay bricks was 40.2%, 52.9%, 61.8%, 68.1%, 71%, and 77.9% lower than the compressive strength of the reference bricks. The reason for this could be the increase in the porosity of the bricks, which is caused by an increase in the amount of marble powder. This in turn results in a decrease of their compressive strength. Vertical and diagonal cracks were formed in the case of applying the maximum compressive load. To evaluate the compressive strength results of the marble powder bricks, an empirical equation describing the compressive strength in relation to the marble powder content was developed. It is shown in Figure 11b. The correlation coefficient (R^2^) is equal to 95%, which confirms the good compliance of the obtained empirical results with the experimental data.

## 5. Comparison of the Experimental Results and Empirical Results

**Figure 12:**
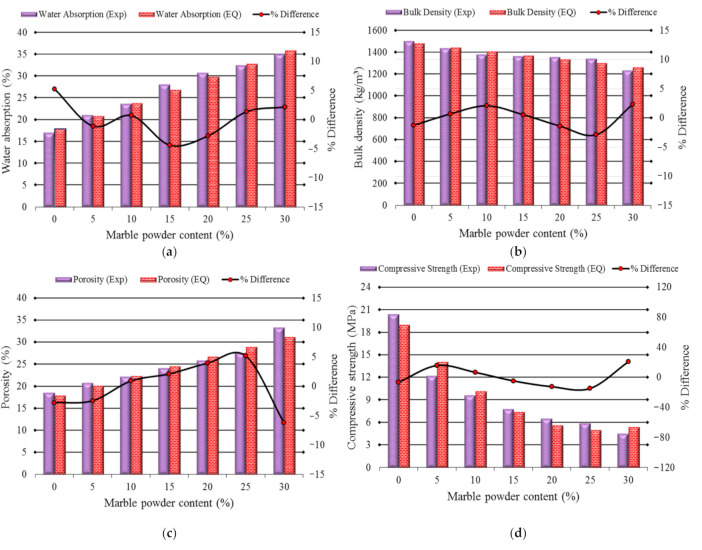
A comparison of the experimental results and those obtained from the laboratory tests for: (**a**) water absorption; (**b**) bulk density; (**c**) porosity (**d**) compressive strength.

To evaluate the characteristics of the marble powder bricks, i.e., water absorption, bulk density, porosity, and compressive strength, the experimental results and empirical results were compared, as shown in Figure 12. Figure 12a shows a graphical comparison of the experimental results and empirical results of the water absorption properties of the bricks. The statistical error/difference ranged from −2.86% to 2.37%. The comparison of the experimental results and empirical results of bulk density is given in Figure 12b. The error ranges from −4.35% to 5.27%. The porosity results of the marble powder bricks, which were obtained experimentally and empirically, are shown in Figure 12c. The error ranges from −6.24% to 5.12%. There is an increasing trend in the obtained results regarding the bricks that have a content of marble powder of up to a 25%. The compressive strength results calculated from the empirical equation were very good. The difference between them and the empirical results presented in Figure 12d ranges from −15% to 20%. Overall, the results obtained from the empirical equations showed very good compatibility with the experimental results, which is confirmed by the level of the maximum error.

## 6. Application of Waste Marble Powder in Eco-Friendly Bricks

### 6.1. Practical Use of the Designed Bricks in the Construction Industry

Marble clay sustainable bricks can be used both in the exteriors and interiors of buildings. In RCC frame structures, marble powder bricks may be used in partition walls due to the fact that such walls do not need to withstand too excessive loads. Depending on the climatic conditions, in some countries, bricks containing waste marble powder can be used for innovative home flooring and for street surface elements. These bricks are easy to recycle, which means they reduce the cost of making new construction materials for new buildings and leave minimal debris behind, in turn reducing the chance of potential pollution. Such bricks can be used in the construction of pavements for pedestrians. According to the results of this work, adding waste marble powder to bricks reduces their compressive strength, which enables them to be used in emergency situations where high strength is not necessary, e.g., in infrastructure facilities such as shelters and temporary hospitals, rooms for construction site workers, or elements of small architecture. For such purposes, a marble clay sustainable brick with a marble powder content of 5% to 20% is suggested for use as a sustainable building material.

### 6.2. Cost Comparison

The price of the raw materials, such as marble powder and clay soil, was analyzed. The cost of transportation is not included in this cost analysis, since it differs regarding the place of production. It was found that the addition of waste marble powder in the manufacturing of bricks is cost effective. The use of waste marble powder in bricks not only reduces the cost of their production, it also contributes to the obtaining of lighter elements, as well as improving their structural performance. The price of the raw materials was given by the different dealers of constructional materials: the marble powder was about USD 0.06 per kilogram, and the clay was about USD 0.09 per kilogram. Therefore, the marble powder was one third cheaper when compared to the clay that was used for the manufacturing of the bricks. Using waste marble powder as a partial substitute for clay reduces the content of the clay, and thus reduces the cost of the bricks. The price of the bricks was reduced by 1.6%, 3.2%, 4.7%, 6.3%, 7.9% and 9.5% with the addition of marble powder of 5%, 10%, 15%, 20%, 25% and 30%, respectively. Table 3 presents a comparison of prices between marble powder and clay per ton quantity (in US dollars).

## 7. Conclusions

Waste materials from marble stone are a major source of concern for stone factories and municipality managements. As a result, the use of waste marble powder as an additive for the production of bricks can improve the natural environment due to the recycling of this waste. For this purpose, the addition of marble powder in different proportions for the manufacturing of bricks was analyzed. The key findings of this study are as follows:

1. The water absorption capacity of the bricks increased with an increase in the content of marble powder.
2. In general, the addition of marble waste to bricks does not significantly affect the amount of salt efflorescence. However, a minor effect of efflorescence was observed when 25% and 30% of marble powder was used in the bricks. The effect of efflorescence was related to the porosity of the bricks, i.e., if the bricks are porous, the effect of efflorescence will be more visible.
3. The bulk density of the bricks declined with a rise in the amount of marble powder, causing the porosity to increase and the weight of the bricks to decrease.
4. The amount of marble powder in clay bricks influences their compressive strength, i.e., as the percentage of marble powder in clay bricks increases, the compressive strength decreases due to increased porosity.
5. The marble powder bricks were lighter in weight than the reference clay bricks. The weight of the bricks decreased as the marble powder content increased. This drop in the weight of the brick samples can result in a large savings for the construction industry, i.e., a higher quantity of bricks and lower structural load.
6. The conducted empirical analysis showed a great compatibility with the laboratory test results regarding the following properties of bricks, i.e., water absorption, bulk density, porosity and compressive strength.
7. Marble clay bricks can be used in emergency situations where high strength is not needed, such as refugee camps, emergency hospitals during flooding and earthquakes, street flooring, and pavements. In such situations, marble clay sustainable bricks with a 5% to 20% marble powder content as are suggested for use as a sustainable construction material.
8. Due to high porosity, the water absorption capacity of marble bricks increased. Therefore, because of this property, it is recommended that marble bricks be used for construction in such areas and countries where the moisture rate in air is relatively low or the bricks are protected against moisture.
9. The use of waste marble powder as a partial replacement for clay in the manufacturing of bricks is cost efficient, as the cost of bricks decreases with the inclusion of marble powder, which has a direct influence on project costs. This study suggests using 5% to 20% waste marble powder as a clay substitute, which reduces the cost of bricks by 1.6% to 6.3%, respectively.

## Figures and Tables

**Table 1:** Proportions of marble powder in the clay bricks.

Brick Groups	Number of Bricks	% of Marble by Weight	% of Clay by Weight	Marble Weight (kg)	Clay Weight (kg)
A	15	0	100	0	50
B1	15	5	95	2.5	47.5
B2	15	10	90	5	45
B3	15	15	85	7.5	42.5
B4	15	20	80	10	40
B5	15	25	75	12.5	37.5
B6	15	30	70	15	35

**Table 2:** Energy dispersive X-ray test report.

Element	Weight %	Atomic %
CK	11.40	18.33
OK	53.66	64.76
MgK	0.25	0.20
CaK	34.69	16.71
Total	100%	99.80%

**Table 3:** Cost comparison of marble powder and clay.

Brick Sample	Marble Powder by Weight %	Clay by Weight %	Marble Powder Price per Ton of Mixture $	Clay Price per Ton of Mixture (USD)	Total Cost of Mix per Ton (USD)
A	0	100	0.0	92.90	92.90
B1	5	95	3.18	88.26	91.44
B2	10	90	6.36	83.61	89.97
B3	15	85	9.54	78.97	88.51
B4	20	80	12.72	74.32	87.04
B5	25	75	15.9	69.70	85.60
B6	30	70	19.08	65.03	84.11

Note: 1 ton of clay = USD 92.9, 1 ton of marble powder = USD 63.6.

## Data Availability

The data presented in this article are available within the article and can be requested from the corresponding authors.
